# Melatonin and Autophagy in Aging-Related Neurodegenerative Diseases

**DOI:** 10.3390/ijms21197174

**Published:** 2020-09-28

**Authors:** Fang Luo, Aaron F. Sandhu, Wiramon Rungratanawanich, George E. Williams, Mohammed Akbar, Shuanhu Zhou, Byoung-Joon Song, Xin Wang

**Affiliations:** 1 Department of Neurosurgery, Brigham and Women’s Hospital, Harvard Medical School, Boston, MA 02115, USA; fluo1@bwh.harvard.edu (F.L.); afsandhu@bwh.harvard.edu (A.F.S.); gwilliams24@bwh.harvard.edu (G.E.W.); 2 Section of Molecular Pharmacology and Toxicology, Laboratory of Membrane Biochemistry and Biophysics, National Institute on Alcohol Abuse and Alcoholism, National Institutes of Health, Bethesda, MD 20892, USA; wiramon.rungratanawanich@nih.gov (W.R.); bj.song@nih.gov (B.-J.S.); 3 Division of Neuroscience & Behavior, National Institute on Alcohol Abuse and Alcoholism, National Institutes of Health, Bethesda, MD 20892, USA; mohammed.akbar@nih.gov; 4 Departments of Orthopedic Surgery, Brigham and Women’s Hospital, Harvard Medical School, Boston, MA 02115, USA; szhou@bwh.harvard.edu

**Keywords:** autophagy, melatonin, neurodegenerative diseases, Alzheimer’s disease, Parkinson’s disease, Huntington’s disease, organophosphate-induced delayed neuropathy, amyotrophic lateral sclerosis

## Abstract

With aging, the nervous system gradually undergoes degeneration. Increased oxidative stress, endoplasmic reticulum stress, mitochondrial dysfunction, and cell death are considered to be common pathophysiological mechanisms of various neurodegenerative diseases (NDDs) such as Alzheimer’s disease (AD), Parkinson’s disease (PD), Huntington’s disease (HD), organophosphate-induced delayed neuropathy (OPIDN), and amyotrophic lateral sclerosis (ALS). Autophagy is a cellular basic metabolic process that degrades the aggregated or misfolded proteins and abnormal organelles in cells. The abnormal regulation of neuronal autophagy is accompanied by the accumulation and deposition of irregular proteins, leading to changes in neuron homeostasis and neurodegeneration. Autophagy exhibits both a protective mechanism and a damage pathway related to programmed cell death. Because of its “double-edged sword”, autophagy plays an important role in neurological damage and NDDs including AD, PD, HD, OPIDN, and ALS. Melatonin is a neuroendocrine hormone mainly synthesized in the pineal gland and exhibits a wide range of biological functions, such as sleep control, regulating circadian rhythm, immune enhancement, metabolism regulation, antioxidant, anti-aging, and anti-tumor effects. It can prevent cell death, reduce inflammation, block calcium channels, etc. In this review, we briefly discuss the neuroprotective role of melatonin against various NDDs via regulating autophagy, which could be a new field for future translational research and clinical studies to discover preventive or therapeutic agents for many NDDs.

## 1. Introduction

Neurodegenerative diseases (NDDs) represent a variety of aging-related non-curable diseases that selectively target different neuron populations in the central nervous system (CNS). Progressive and chronic aggregations of unique proteins in the organs such as the brain and spinal cord are hallmarks of diverse NDDs. Due to the inability to get rid of the irregular aggregate proteins, neurons are unable to properly function and begin to degenerate. NDDs are a series of diseases that negatively affect the nervous system and cause neurological dysfunction in the patients with damaged nerve cells [1,2]. Common age-related NDDs include Alzheimer’s Disease (AD), Parkinson’s disease (PD), Huntington’s disease (HD), and amyotrophic lateral sclerosis (ALS). The etiology and pathogenesis of these NDDs are complex and unique for each disease. Genetic mutations and environmental factors such as excessive alcohol consumption, smoking, and aging play contributing roles in the etiology and pathogenesis of various NDDs [3]. Most of NDDs progress slowly with a positive correlation between prevalence and aging. These NDDs can last for decades and can be affected by both genetic factors and environmental triggers [4].

Classical cell death mechanisms, i.e., necrosis or apoptosis, exist in many forms of NDD. Recent studies have shown that certain proteins in neural tissues and nerve cells of NDDs were abnormally increased and produced aggregates of abnormal proteins with potential toxicities, resulting in gradual loss of physiological functions of nerve cells, and triggering cell death [5,6]. Autophagy is a catabolic process to degrade the cytoplasmic components and remove dysfunctional components, such as damaged organelles and proteins [7,8]. It is closely linked with cell growth, development, and various diseases, depending on the cellular contexts, environments, and exposures to certain agents. Autophagy relies on autophagic lysosomes to engulf and degrade damaged organelles, and abnormal proteins to maintain cell homeostasis. Impaired autophagy in humans leads to the aggregation and accumulation of beta-amyloid (Aβ), cytoskeleton-related protein tau, and β-synuclein in neuronal cells and tissues, even though the contributing role of these aggregated proteins is not well understood in the specific etiology of each NDD. While larger protein aggregates are generally known to be toxic to the cells, other studies demonstrated that smaller aggregates such as Aβ oligomers can also cause damage in certain forms of NDD [9]. However, the cellular levels of these aggregates correlate positively with the severity of the disease [10,11]. At present, the mechanisms of cell death in NDDs are still not fully understood. Furthermore, there are no effective treatments to cure or prevent these aging-related NDDs, although some treatments may attenuate certain symptoms. Thus, to explore the roles of autophagy and identify protective factors in various NDDs is of great significance for early diagnosis, prevention, and development of effective therapeutic methods.

## 2. Neuronal Death in NDDs

### 2.1. Cell Death

Cell death is usually divided into “necrosis” and “programmed cell death or apoptosis”. Traditionally, “necrosis” is used to explain a variety of cell deaths, i.e., different physiological responses of cells when cells are exposed to different external toxicants. The necrosis process is fierce and can quickly cause cells to lose their primary function, ultimately rendering the cells unable to maintain internal stability [12]. Unlike necrosis, the main function of programmed death is to remove “non-essential” cells that are no longer needed, such as the removal of some cells at a particular stage of development. Apoptosis, the programmed cell death, is an active process that requires gene transcription and expression and a very common phenomenon in the development of vertebrates and invertebrates.

Necrosis is the irreversible death of a cell when it is fatally damaged, usually after acute exposure to certain toxic agents. The characteristics of necrosis are as follows: (1) Loss of membrane integrity of necrosis cells, (2) starting from cytoplasmic and mitochondrial swelling, ending with complete cell lysis, (3) no vesicle formation in the cells, (4) loss of regulation of ion homeostasis, (5) no energy requirement, (6) random digestion of DNA, (7) caused by non-physiological agents and affects a variety of cells, and (8) usually accompanied by phagocytosis of cell debris and inflammatory response of macrophages [13,14,15].

Apoptosis is a normal and important process of self-correction in the development of various organs of the body. Through a natural cell death program, excess cells can be removed, cells with abnormal phenotypes can be cleared, errors in development can be corrected, and functions between various systems can be more coordinated. It was shown that the apoptotic pathway is activated when certain cells are no longer needed or damaged [16]. Moreover, the occurrence of apoptosis was also related to the cell type, maturity, and developmental stage [17,18,19]. The characteristics of apoptosis are as follows: (1) Apoptotic cell membranes bleb, but retain integrity, (2) beginning with the shrinking of cytoplasm and condensation of the nucleus, (3) formation of apoptotic bodies, (4) tightly regulated process in the activation of the proteins involved in apoptosis, (5) ATP-dependent, (6) non-random mono- and oligonucleosomal length fragmentation of DNA, (7) activation of the caspase cascades, (8) affecting individual cell, (9) induced by physiological stimuli, (10) phagocytosis by adjacent cells or macrophages, and (11) no inflammatory response [13,14]. Consequently, apoptosis usually takes place to conserve energy and resources by purging unnecessary cells that are impractical for the biological system.

### 2.2. Autophagy

Cell autophagy is a process in which lysosomes remove aging-related damaged organelles, misfolded proteins, and intracellular pathogens, and reuse cellular components to obtain cell stability and save energy [20,21]. Under the stimulation of harmful factors in the internal and external environments, autophagy is activated in response to the stress of the cell. It renews the organelle and carries out cell metabolism, maintains cellular homeostasis, and plays an important role in the development, differentiation, and senescence [20,21,22,23,24,25].

#### 2.2.1. Autophagy Classification

There are three types of autophagy: Microautophagy, macroautophagy, and chaperone-mediated autophagy. Macroautophagy is composed of intracellular and endoplasmic reticulum-derived bilayer membranes that envelope cytoplasmic proteins and organelles and combine with lysosomes to form autophagolysosomes, which serve as substrates for degradation by lysosomal hydrolases [23,26,27]. Macroautophagy is commonly known as autophagy. Microautophagy is the deformation of the lysosomal membrane, which directly encapsulates the irregular or damaged components in the cytoplasm. Inside the lysosomes is an acidic environment with hydrolytic enzymes to degrade abnormal organelles and macromolecules into their subunits so that the cell can reuse or allocate them elsewhere. Molecular chaperone-mediated autophagy is the combination of molecular chaperone-substrate complexes by specific molecular chaperones in the cytoplasm, which then bind to proteins on the lysosomal membrane and translocate the substrates into the lysosome for their degradation [20,27]. Chaperone-mediated autophagy requires specific “chaperones” to recognize and bind the target substrate molecules before they can be transported to lysosomes for degradation. These degraded substrates require specific amino acid sequences and are recognized and bound by “molecular chaperones”. Therefore, this type of autophagy is highly selective and generally responsible for the degradation of only proteins, but not organelles [28].

#### 2.2.2. The Molecular Mechanism and Process of Autophagy

Nearly 40 autophagy-related genes (ATG) have been identified and 15 genes are known to encode autophagy proteins, which regulate the autophagy process [29]. ATG protein complexes mainly consist of the following types: (1) Unc-51-like kinase 1 (ULK1) complex, consisting of ULK1, ATG13, FIFP200, and ATG101; (2) type III phosphatidylinositol 3-kinase (PI3K-III), consisting of ATG14L (ATG14-like), Vps34 (type III PI3K), Vps15, and Beclin1 (Bcl2 interacting protein); (3) ATG- WIPI; (4) ATG9; (5) ATG5-ATG10-ATG12 system; and (6) ubiquitin-like binding protein LC3-II [25,29,30,31,32,33] (Table 1).

The ATG protein complex activates and participates in various signaling pathways to complete the regulation of the entire autophagy. The autophagy process is divided into several stages: (1) Induction, (2) vesicle nucleation, (3) vesicle elongation, and (4) fusion and degradation. Autophagy-related proteins catalyze specific reactions at each step and are essential for the production of autophagic fluxes [20,62,63,64].

Induction: In autophagy research, the most frequently used induction methods are starvation or nutritional deficiencies, while ethanol-induced autophagy can also be employed [65]. Starvation or alcohol-induced autophagy usually relies on the inhibition of mammalian rapamycin target protein (mTOR), which is a key regulator of nutritional signals. In mTOR inhibition, the phosphorylation of protein complexes, including ULK1, ATG13, and RB1-coiled helix-1, is simultaneously reduced, increasing ULK1 activity and inducing autophagy [66,67,68,69,70].

Vesicle nucleation: Membranes of newly formed phagocytic vesicles are originated from the Golgi apparatus, ER, mitochondria, or endosomes. The formation of primitive phagocytic organelles or phagocytic vesicles depends on the release of Beclin1 and activating molecules present in Beclin1-regulated autophagy protein 1 from the inhibitors Bcl-2. The resulting PI3K-III complex is released from the inhibition, and this complex produces phosphatidylinosine-3-phosphate and promotes the recruitment of ATG proteins into the nascent membrane [69,71,72].

Vesicle elongation: Activation of two ubiquitin-like conjugates promotes vesicle extension. In the first complex, ATG7 and ATG10 participate in the covalent binding of ATG12 and ATG5. The interaction of ATG12/ATG5 covalent binding complex with ATG16 can promote the formation of the second ubiquitin-like conjugate complex. In the second complex, ATG4, ATG7, and ATG3 are involved in the conversion of soluble LC3 into lipid conjugate LC3-II. LC3 is a phosphatidylethanolamine-based lipid associated with phagocytic membranes. These two ubiquitin-like conjugates are therefore involved in the formation of autophagosomes. At the end of the extension phase, the phagocytic vesicles have expanded and then closed, completing the isolation of the intracellular components. All autophagy-associated proteins leave the autophagosomes and return to the cytoplasm. The lipidated LC3 is the only autophagy-related protein believed to be involved in autophagosome membranes [29,43,73,74].

Fusion and Degradation: When starved, the SNARE protein synaptic fusion protein-17 (STX17) is located on the autophagosome. The interaction of STX17, SNAP29, and lysosomal VAMP8 causes autophagosomes to fuse with lysosomes and form mature hydrolyzed organelles. After fusion, proton pumps and hydrolase enzymes are released into the autophagosome, forming an autophagolysosome, which acidifies and hydrolyzes the cell contents [75,76].

Autophagy removes damaged intracellular organelles, accumulated and misfolded or aggregated proteins, and other substances for subcellular level reconstruction [24]. At the same time, autophagy, as an adaptive catabolic pathway, can respond to different forms of stress such as hypoxia and nutritional deficiencies. The products released from its decomposition pathway or cycle enter the tricarboxylic acid cycle, an important set of reactions for the generation of high-energy-carrying molecules to be used for proper supply of cellular energy. Autophagy as a regulator of DNA damage response also regulates cell apoptosis and death [77]. Under normal conditions, autophagy is maintained at a low level. It is a conservative metabolic system and a cell-protective mechanism, which can maintain intracellular homeostasis and regulate cell behavior [24].

## 3. Autophagy and NDDs

NDDs are chronic progressive and aging-associated diseases. They are usually caused by the misfolding and the accumulation of some specific proteins in the nervous system often observed in aged people and living mammals. Accumulated misfolded proteins usually stimulate the death of neuronal cells, eventually contributing to a type of disease in which patients exhibit unique signs of neurological dysfunction and symptoms specific to each NDD. They are characterized by the degeneration or damage of nerve cells frequently accompanied by increased oxidative stress, ER stress, mitochondrial dysfunction, and neuroinflammation [78,79].

NDDs have complex etiology and pathogenesis mechanisms. In recent years, scientists in numerous laboratories have studied the role of autophagy in NDDs and showed that lysosomal autophagy and ubiquitin-proteasome pathways might be the major mechanisms to clear misfolded and abnormal proteins [80,81]. In addition, autophagic degradation of aggregated proteins was associated with decreased amounts of aggregated proteins and reduced toxicity [82]. Impairment of these pathways might reduce the cellular ability in removing the damaged and/or aggregated proteins, and various mutations of autophagy receptors or pathways were shown to be related to NDDs [59,83]. In this connection, we have reviewed the results of recent studies on the association between autophagy and various NDDs including AD, PD, HD, OPIDN, and ALS.

### 3.1. Alzheimer’s Disease (AD)

AD is common in elderly people and may begin from middle-age in some individuals, especially with mutations in AD-associated genes. The main pathological characteristics of AD are the deposits of amyloid-β (Aβ) fragments produced from amyloid precursor protein (APP), the presence of senile plaques (SP) with neurofibrillary tangles caused by excessive phosphorylation of tau proteins in nerve cells, and functional impairment or loss of hippocampal and cortical neurons [84]. It is not completely understood how aggregated Aβ oligomers cause cytotoxicity and/or cell death. In a cell culture model system, Friedrich et al. reported a potential mechanism of an intracellular basis of Aβ-related pathogenesis [85]. In this model, a soluble, single amyloid plaque, which may be present outside of the cell or be produced extracellularly, can be internalized via receptor-mediated endocytosis or phagocytosis and then become incorporated into multivesicular bodies that grow bigger in size and penetrate the vesicular membranes. This process of larger Aβ plaque aggregates can cause cell death with subsequent release of amyloid plaques into the extracellular space for greater cytotoxicity. In addition, Aβ aggregation can cause local neuroinflammation, oxidative stress, activation of mitogen activated protein kinases (MAPKs), and tau protein phosphorylation [86,87], contributing to neuronal death.

Animal models and human studies have shown that Aβ-related oligomers and aggregate proteins involved in the pathological process of AD are closely maintained by the two major systems of lysosomal autophagy pathways and ubiquitin-dependent proteasomes, as detailed by [88]. For instance, most extracellular proteins, including amyloid plaques, can be internalized through the receptor-mediated endocytosis [85] and likely degraded in the lysosomes, since these organelles contain acid proteases and acid hydrolases [88]. In addition, other enzymes, such as insulin-degrading enzymes and matrix metalloproteinases, could be released into the extracellular space and thus degrade Aβ to prevent its deposits [88]. Additionally, a metalloprotease can prevent intracellular Aβ release into the circulating exosomes, as recently reported [89]. Recent studies have revealed that part of the secreted enzyme complex is presenilin (PS), which is an essential protein for lysosomal acidification and removal of APP, and it was, therefore, related to autophagy [90,91]. PS1 is involved in the glycosylation and transport of lysosomal H^+^-ATPase (V-ATPase). The PS1 mutant (mPS1) results in impaired lysosomal acidification and reduced autophagic degradation. In fact, genetic mutations of PS1 and PS2 are associated with the pathogenesis of AD as demonstrated in the familial autosomal dominant AD [92,93,94,95].

Recent studies have shown that the phosphatidylinositol-binding clathrin assembly protein (PICALM) is involved in tau autophagy and clearance [96,97]. PICALM has also been found to be involved in autophagy in vivo and in vitro, including elongation of phagocytes, formation of autophagosomes, and fusion between autophagosomes with lysosomes [98,99]. A decrease or disappearance of PICALM in the brain of AD individuals revealed that it might be associated with an increased risk of AD. Other studies have shown that reduced Beclin1 in mouse models could lead to the accumulation of Aβ and neuronal degeneration. These results with experimental models also confirmed that neurons in AD patients have decreased Beclin1 expression, suggesting that the synthesis of autophagosomes may be delayed or even inhibited [100,101]. Some studies have shown that the reduction of Beclin1 decreased the degree of autophagy with increased Aβ deposition in neuronal cells, suggesting an important role of autophagy in AD pathogenesis in AD mouse models constructed with the APP transgene [102,103].

The mammalian target of rapamycin (mTOR) regulates autophagy. The use of rapamycin or starvation could inhibit mTOR activity and thereby increase autophagy activity [104,105]. In in vitro and rodent AD model studies, mTOR activity was correlated with Aβ accumulation. In animal models of AD, the use of drugs to inhibit mTOR activity could reduce the cognitive decline due to Aβ accumulation [106,107,108,109]. Studies with the brains of AD patients have shown that high mTOR activity was also associated with tau hyperphosphorylation and elevated neurofibrillary tangles [110,111,112,113]. NRBF2 is a key component and the regulator of PI3K-III (the class III phosphatidylinositol 3-kinase). Yang et al. reported that the expression of the autophagy substrate SQSTM1 was increased in cells with over-expressed NRBF2, suggesting that decreased NRBF2 impairs the degradation of SQSTM1, indicating that NRBF2 is involved in the autophagy regulation. The amount of NRBF2 protein in the hippocampus of 5X-FAD dementia mouse models was also reduced. Moreover, overexpression of NRBF2 reduced the amyloidosis of APP, and inhibition of NRBF2 could increase the amyloidosis of APP in AD cell models. These results indicated that there is a close interaction between NRBF2 and APP by regulating the rate of autophagy [114]. A variety of cytosolic components were degraded in mitochondria, protein aggregates, and peroxisomes via the autophagy receptor p62/SQSTM1 [60,115]. The amount of p62/SQSTM1 was inversely associated with the rates of clearance of Aβ and hyperphosphorylated tau [116,117]. Studies have found abnormal levels of phosphorylated p62/SQSTM1 in AD brains and these inactivated proteins were positively associated with neurofibrillary tangles [118,119]. All of these facts strongly indicate that autophagy plays a critical role in the etiology and pathogenesis in AD.

### 3.2. Parkinson’s Disease (PD)

PD is mainly affected by genetic and environmental factors, and its exact etiology is unknown, although increased oxidative stress and mitochondrial dysfunction are involved [120]. PD is mainly caused by the reduction of dopaminergic neurons in the nigrostriatal extracorporeal system of the midbrain. The main functions of the dopaminergic neurons of the nigrostriatal body are to regulate autonomous movements, maintain posture, and coordinate gait. Due to the degeneration of the dopaminergic neurons, affected people show signs of muscle stiffness, resting tremors, and posture instability; other pathways involved in sleep, cognition, mental abnormalities, and other non-motor symptoms are also affected [121,122,123]. The main pathological features of PD are the reduction of dopaminergic neurons in the extrapyramidal nigrostriatal body and the formation of Lewy bodies formed by the aggregation of α-synuclein and its oligomers surrounded by neurofilaments. Normally, α-synuclein is encoded by multiple genes on the autosome and is responsible for regulating dopamine metabolism. When this gene is mutated, it can cause the pathological accumulation of α-synuclein to form Lewy bodies and stimulate neuronal degeneration [124,125,126,127]. In addition to genetic mutations of α-synuclein gene, increased post-translational modifications of this protein under elevated oxidative stress in PD could result in the accumulation and/or aggregation of its oligomers [120]. Even before a person shows typical signs of symptoms associated with PD, dopamine levels are reduced, and dopaminergic neurons have started to degenerate. Studies have shown that at the time of symptoms present, more than 50% of dopaminergic neurons were lost and cellular levels of dopamine were reduced by more than 70% [128].

It was shown that α-synuclein is degraded by chaperone-mediated autophagy (CMA) and macroautophagy pathways, where CMA is the main mechanism [129,130]. However, mutated α-synuclein leads to its decreased binding to chaperone protein molecules in CMA, leading to the formation of Lewy bodies [124,126]. Some cases of familial PD were related to the repeated expression of the α-synuclein gene. Both in vivo and in vitro experiments have confirmed that abnormally increased expression of α-synuclein resulted in decreased macroautophagy [131,132]. The dislocation of ATG9 transmembrane protein involved in the initial membrane precursor formation of phagocytic cells was related to this change [133]. Kam et al. reported that the α-synuclein could activate the poly-ADP ribose polymerase 1 (PARP-1), a protein important in DNA repair, genomic instability, and programmed cell death, which promoted the formation of the pathological α-synuclein and eventually led to cell death [134]. In PD patients, elevated levels of cleaved PARP were found in cerebrospinal fluids and brains. This indicates a direct involvement of PARP in PD pathogenesis. The facts that the PARP RNA interference knockdown or PRP inhibitor 3-aminobenzamidereduces the LC3-II accrual and the autophagic inhibitor 3-methyladenine inhibits C42-induced cleavage of PARP suggest that PARP may be related to autophagy regulation [135]. Moreover, recent studies have revealed that PARP-1 inhibited autophagy through the AMPK/mTOR pathway [136,137]. Using PARP inhibitors or knocking out the PARP genes could effectively prevent the toxicity of pathological α-synuclein, suggesting that the PARP-1 activation played an important role in the pathological changes of PD [137,138]. Mutations in PARP-1 and α-synuclein-related genes have been shown to be associated with the occurrence of PD. In autosomal dominant PD, *SNCA* (encodes α-synuclein), *LRRK2* (encodes leucine repeat kinase 2 involved in autophagy and other processes), *VPS35* (encodes endosomes and vacuolar protein 35 in vesicles), *DNAJC13* (encodes REM-8), and *CHCHD2* (encodes mitochondrial protein) were mutated. Among them, *LRRK2* mutation was the most common cause of delayed-onset PD [112,139]. In autosomal recessive PD, genes for Parkin (E3 ubiquitin ligase), PINK1 (PTEN-induced kinase 1), and DJ-1 (Park7, an antioxidant defensive enzyme and deglycosylase) are mutated and related to the occurrence of early-onset PD, and Parkin mutations are more frequent. Studies have found that treatment with mitochondria-damaging agents such as doxorubicin- and cisplatin-activated Parkin, which then promoted the ubiquitination of various mitochondrial proteins, induced autophagy, and cleared damaged mitochondria. It is also reported that Parkin deficiency exacerbated alcohol-induced dopaminergic neurodegeneration in a mouse model of PD through P38 pathway-dependent inhibition of autophagy and mitochondrial function [140]. The PINK1 gene can mediate autophagy. Studies have shown that PINK1 and Parkin played a role in promoting mitochondrial autophagy. Mutations in Parkin and PINK1 genes result in the defective mitochondria and lead to the pathogenesis of early-onset PD [141,142]. Mutations in the E3 ubiquitin ligase Parkin and the protein kinase PINK1 are linked to autosomal-recessive juvenile Parkinsonism (AR-JP). These mutations cause defects in mitophagy. Parkin requires activation by PINK1 and PINK1 can phosphorylate the ubiquitin and the parkin ubiquitin-like (Ubl) domain. Parkin binds phospho-ubiquitin, which led to efficient parkin phosphorylation [143]. Other autophagy-related gene mutations that occur less frequently include *ATP13A2* (involved in the regulation of autophagy), *GBA* (glucocerebrosidase β acid, involved in the regulation of autophagy), and *SCARB2* (encodes LIMP-2 with an important role in regulating lysosomal/autophagosomes function) [122,144,145].

Methyl-4-phenyl-1,2,3,6-tetrahydropyridine (MPTP), which is the byproduct of making 1-methyl-4-phenyl-4-propionoxypiperidine (MPPP), can stimulate selective death of the substantia nigra neuron population. Astrocytes oxidize this molecule MPTP into 1-methyl-4-phenylpyridinium (MPP^+^), which inhibits complex I in the electron transport chain in mitochondria. As a major inhibitor of autophagy, mTOR has been found to block MPTP-induced neuronal death. Neuronal death was not only observed in PD mice, but also accompanied by certain adverse reactions, such as immunosuppression, leukopenia, and respiratory infection. Thus, AMPK-mTOR-mediated autophagy and apoptosis signaling pathways might be potential targets for treating PD [146,147,148,149]. Other studies have shown that transfection of the stable Beclin1 virus plasmid into cells activated autophagy, reduced the accumulation of protein aggregates, and decreased the incidence of PD [148,150].

### 3.3. Huntington Disease (HD)

HD is an NDD that occurs in the striatum and cerebral cortex. It is also known as hereditary chorea. Its occurrence is mainly due to the mutation of the *Huntingtin* (*HTT)* gene in the chromosome 4, which results in the accumulation of mutant Htt proteins (mHTT) that form clusters, and affects nervous system function. The importance of the polyglutamine tract length as a determinant of disease severity has been reported in animal models of HD [151]. Its characteristics include involuntary movements, progressive dementia, mental disorders, such as chorea, ataxia, depression, and personality disorders [152,153].

The normal HTT protein is extremely important for the development of neurons, but the gene responsible for HTT is sometimes mutated to express the mHTT with an aggregation tendency to damage the CNS [154]. Recent studies have shown that dopamine D2/D3 receptor activators could induce autophagy, thereby eliminating accumulated soluble mHTT. The D2/D3 receptors were expressed in the striatum, and D3R played a major role in the pathogenesis of HD. Luis et al. [155] treated R6/1 mice (transgenic HD mouse model) with pramipexole (PPX, a D2/D3 receptor activator) for 4 weeks and found that PPX reduced the level of soluble mHTT in the striatum and protected striatum neurons, suggesting that these changes were D3R-dependent. These beneficial effects were mediated by the activation of autophagy by PPX treatment. In this mouse model, it was also found that PPX increased the number of autophagosomes and that the possibility of transcriptional suppression of proteasome activation was ruled out. Autophagy could not remove all mHTT expressed in all brain cells. Although autophagosomes in HD cells mature normally, they are nonfunctional [156]. Further studies have shown that HTT could competitively bind to ULK1, antagonize the mTOR pathway and attenuate the inhibitory effect of mTOR in the initial phase of autophagy [157,158]. The natural compound quaternary ammonium berberine-like isoquinoline alkaloids have been found to prevent the accumulation of mHTT to activate autophagy in HD cells and mouse models, thereby alleviating symptoms of HD [159].

A large number of phagocytic cells were found in the brain tissues of HD patients and experimental animals. The main mechanism might be the non-specific binding of aggregated mHTT and Beclin1, leading to the consumption of Beclin1, and thereby decrement of the number of autophagosomes. Recent reports have shown that reduced expression of the autophagy protein Beclin1 resulted in reduced autophagy levels that also caused aggregation of mHTT [160]. Autophagy receptor p62/SQSTM1 and autophagy-related FYVE protein (ALFY) played important roles in mHTT degradation [156,161]. Consistently, ALFY mRNA expression was reduced in the brains of HD patients [162,163,164]. Small activator molecules of autophagy can remove the mHTT protein in different HD models including yeast, fly, and mammals [165]. Despite numerous studies, the protective mechanisms of autophagy in HD have not been fully understood and further investigation is needed.

### 3.4. Organophosphate-Induced Delayed Neuropathy (OPIDN)

OPIDN is an irreversible degenerative nerve disease that gradually appears about 7 to 14 days after poisoning with various insecticides or pesticides. It is mainly manifested as flaccid paralysis, spinal cord injury, and ataxia. OPIDN is an NDD after exposure to toxic, poisonous substances such as organophosphate-containing pesticides. In OPIDN, the degeneration and accumulation of irregular mitochondria, vesicles, and nerve fibers may be caused by abnormal autophagy. Dysregulated degradation and the aberrant accumulation of damaged or swollen mitochondria, vesicles, and irregular nerve fibers are seen in many neurons [166,167,168]. Organophosphorus compounds, including tri-orthocresyl phosphate (TOCP), induce OPIDN symptoms. Studies have also shown that the symptoms of OPIDN caused by TOCP were associated with significant changes in the autophagy-related protein Beclin1 in the nervous tissues in adult hens, and levels of severity of this disease were negatively correlated with functional autophagy levels [169]. Studies have shown that exposure to TOCP and other organophosphorus compounds could induce OPIDN symptoms in adult hens, and these symptoms were inversely correlated with autophagy levels. Other studies have shown that TOCP could cause delayed neurotoxicity in humans and sensitive animals, and its potent neurotoxicity was also related to decreased autophagy. These findings suggested that the autophagy regulation process in the nervous system might be involved in the pathogenesis of OPIDN [169,170,171].

### 3.5. Amyotrophic Lateral Sclerosis (ALS)

Studies have shown that a series of genetic mutations are associated with ALS [172,173]. Mutations such as the Cu/Zn-dependent superoxide dismutase 1 (SOD1) gene caused loss of dynein function, leading to the accumulation of pathological protein aggregates in ALS [174,175,176,177]. Studies have shown that genetic mutations enhanced the binding of autophagy matrix to autophagosomes through interaction with LC3 [178,179,180]. Pathogenic mutations in ubiquitinated autophagy receptor protein optineurin (OPTN) reduced autophagy with less protein clearance and inhibited the OPTN’s ability to remove damaged mitochondria [181,182]. Abnormal protein aggregation of mutant SOD1 is one of the main causes of progressive loss of motor neurons in ALS. As a gene that regulates autophagy, transcription factor EB (TFEB) promotes autophagy by enhancing Beclin1 expression. TFEB overexpression increased the proliferation and survival of neural cells and animal modes of ALS with SOD1 mutations [183] (Table 2).

## 4. Translational Applications of Melatonin against NDDs

### 4.1. Melatonin

Melatonin was first isolated and extracted by Lerner et al. in 1958. It is a fat-soluble indole pineal hormone with a molecular weight of 232.3 Da and a half-life of about 10 min [184]. Melatonin is mainly secreted by the pineal gland in mammals, but it may also be produced by non-pineal cells like retina, bone marrow, and gut. Human melatonin secretion is generally decreased with increased age. The human pineal gland secretes melatonin from 3 months after birth, peaks at 6 years old, and decreases to adult levels at 12 years old. The pineal gland is then calcified around the age of 16, and melatonin levels in the blood are inversely associated with increased age. Under normal physiological conditions, the concentration of melatonin in humans and other mammals fluctuates with the circadian rhythms, showing that the peak secretion at night and the diurnal drop to the valley value are regular cyclic changes related to environmental lighting conditions [184,185,186]. Melatonin, as an internal clock molecule with antioxidant activity, has been used widely for helping sleep better and/or improving jet-lag conditions.

Elderly people often have biological rhythm disorders accompanied by poor sleep at night, and the responsiveness and cognitive decline during the daytime. Magri’s research showed that with increasing age, the peak of serum melatonin secretion at night and the total amount of secretion for 24 h decreased. Moreover, the diurnal fluctuation was low, especially in elderly people with dementia symptoms, and its level was significantly and negatively correlated with the degree of cognitive function. That is, the worse the cognitive ability, the lower the level of melatonin [187]. Schernhammer et al. reported that night shift workers have relatively lower melatonin levels with higher risks of cancer [188]. In fact, Skene has reported that melatonin affects the circadian rhythm, sleep quality, sexual maturity, reproduction, immunity, oxidative stress, and tumor incidences even in blind people with non-24-h sleep/wake disorder [189].

### 4.2. Molecular Mechanisms of Protection by Melatonin against NDDs

#### 4.2.1. The Effects of Melatonin on Autophagy

Under certain brain pathological conditions, such as abnormal protein aggregation, neurotoxic agent (e.g., methamphetamine, MPTP, kainic acid, rotenone)-induced autophagy can lead to pathological changes, including ER stress and mitochondrial dysfunction, and stimulate cell death. Melatonin can prevent autophagy and protect neurodegeneration. Melatonin promotes basal levels of autophagy under physiological conditions, thereby maintaining the homeostasis and survival of neurons. The effect of melatonin on autophagy might be related to the level of activation of catabolic processes and specific cellular conditions [190].

Previous studies have revealed that melatonin has a protective effect on AD, and could reduce the production of amyloid plaques in N2a/APP cells possibly through its antioxidant property [191,192]. Melatonin can increase autophagy and reduce amyloid production partly through the regulation of reactive oxygen species (ROS) and inflammation [191]. The uncontrolled, continued production of ROS in cells could damage mitochondrial proteins, lipids, and DNA, resulting in increased autophagy [193]. Recent studies showed that both mitochondria and ER play a key role in the formation of autophagosomes [194,195]. It was well-established that melatonin could regulate many targets involved in the ER stress process such as increasing the Bcl-2/Bax ratio, restoring catalase, Cu/Zn-SOD, Mn-SOD, and COX-2. Through this interaction, melatonin could affect autophagy and apoptosis [196]. Melatonin exhibits a beneficial effect on mitochondria by reducing the oxygen consumption rate, oxygen flux, membrane potential, and ultimately suppressing the production of superoxide anions and hydrogen peroxide [190]. Research by Scheper et al. stated that in an NDD like AD, the unfolded protein response (UPR) was related to tau protein aggregation as well as impaired autophagy process in the brain of each AD patient [197]. Melatonin treatment could restore the autophagy flux, thereby preventing tauopathy and cognitive decline in AD mice [198]. Other studies showed evidence that melatonin exhibited a beneficial effect on AD symptoms possibly through the restoring the mitochondrial function and autophagy, but more research is needed to study the effects of melatonin on AD brain autophagy [15,199].

PD is the second most common NDD. Degeneration of dopaminergic neurons and aggregation of α-synuclein in the nigrostriatal region of the brain are the main pathological manifestations of PD, but the mechanism for selective destruction of these regions are still unclear.

A few reports have shown that melatonin exerts protective effects in several experimental models of PD [200,201,202,203]. For a long time, a chemical, 1-methyl-4-phenyl-1,2,3,6-tetrahydropyridine (MPTP), has been used as a tool to induce PD in animal models [204], since it is a neurotoxic and psychoactive agent [205]. Su et al. used MPTP to selectively destroy dopaminergic neurons in the substantia nigra [204]. MPTP increased the activity of cyclin-dependent kinase 5 (CDK5) in the mouse model of PD. CDK5 was shown to be involved in the regulation of autophagy to mediate neuronal cell death and neurotoxicity [206,207,208,209]. Studies reported that MPTP increased LC3-II in C6 glioma cells and mouse striatum cells through CDK5 activation [204].

Melatonin could reduce MPTP-induced α-synuclein aggregation in mice. Furthermore, melatonin pretreatment reduced MPTP-induced loss of axon and dendritic length in dopaminergic neurons through suppression of autophagy activated by CDK5 and α-synuclein aggregation, thereby reducing dyskinesia symptoms in PD animal models [204,210,211,212]. Melatonin also reversed the increased levels of LC3-II and the decreased SQSTM1 levels, while it reduced MPTP-induced autophagosome formation [213,214,215].

Kainic acid has also been used to induce PD in experimental models [216]. One study showed that it increased α-synuclein and LC3-II levels in the hippocampus of mice with neuronal loss. Melatonin enhanced the ubiquitination of α-synuclein and reduced LC3-II, cathepsin B, and lysosomal-associated membrane protein 2 (LAMP-2) [217]. Melatonin attenuated kainic acid-induced neurotoxicity by inhibiting autophagy. Zhou et al. used rotenone to induce degeneration of dopaminergic neurons. This study showed that rotenone promoted autophagic cell death, and its toxic effect was mediated by the release of Bax and Omi into the cytoplasm [218]. Pretreatment with melatonin reduced cell death by reducing Bax expression and the release of Omi into the cytoplasm. Melatonin could inhibit the activation of c-Jun N-terminal protein kinase 1, JNK, the upstream cell death pathway kinase, through phosphorylation of pro-apoptotic Bax prior to its translocation to mitochondria for promoting mitochondria-dependent apoptosis [219], thus protecting cells from autophagic cell death triggered by the Bcl-2/Beclin1 pathway. These results provided additional information on the role of melatonin in regulating apoptosis and autophagy signaling [220]. Other studies have shown that E3 ubiquitin ligase Parkin protein was lost in PD patients [221]. Parkin could cause autophagy-mediated degradation of dysfunctional mitochondria in the affected cells, suggesting that PD might be linked to the inability to eliminate dysfunctional swollen mitochondria [222]. Parkin’s translocation to mitochondria required the protein kinase PINK1, which was located in the mitochondrial membrane of the human brain. Co-expression of Parkin and PINK1 enabled mitochondrial aggregation to form autophagic vesicles. Parkin or PINK1 mutations caused mitochondrial regeneration disorders that could cause defective mitochondrial accumulation and neurodegenerative changes frequently observed in PD. Overexpression of Parkin enhanced mitochondrial autophagy (mitophagy). Recently, other studies showed that PINK1 recruited Parkin to depolarize mitochondria [223,224]. After Parkin was recruited to the mitochondrial membrane, it stimulated the formation of Lys27 polyubiquitin chains on VDAC1 and the recruitment of a phage p62 complex. In addition, Lys63 polyubiquitin chains also played an important role in delivering Parkin-labeled mitochondria to autophagosomes. Therefore, both types of ubiquitin linkage, might transport pro-apoptotic factors to the lysosomes through the autophagy pathway [225]. Therefore, Parkin was able to direct damaged mitochondria to autophagosomes to prevent them from releasing pro-apoptotic factors, thus establishing a link between the two pathways [226]. Mitochondrial autophagy induced by PINK1 and Parkin played an important role in maintaining the healthy mitochondrial network and helped reduce neural damage caused by mitochondrial dysfunction in PD [221].

The neurotoxicity of methamphetamine (METH) can cause neurological symptoms similar to PD [205]. When METH induced autophagic cell death in dopaminergic cell lines, it disturbed the Bcl-2/Beclin1 complex. Melatonin pretreatment stabilized the Bcl-2/Beclin1 complex, blocked autophagy, and protected cells from autophagic death. Therefore, it is likely that melatonin protects cells from autophagic cell death triggered by the Bcl-2/Beclin1 pathway through inhibition of JNK1 (c-Jun N-terminal kinase 1) and activation of Bcl-2 upstream pathway [215]. Melatonin also activated mTOR, which inhibited METH-induced autophagy [227].

Prion disease is another type of NDDs. During infection, viral proteins (PrP) induce misfolding of normal cellular proteins. PrP-mediated neurodegeneration exhibited similar characteristics of misfolded aggregated proteins, such as Aβ, α-synuclein, and tau, often associated with NDDs [228]. Similarities between these proteins include conformational diversity, movement of aggregates across cells, and the proliferation of aggregate proteins in the affected cells. Studies have found that mitochondrial dysfunction of SH-SY5Y neuroblastoma cells exposed to PrP eventually ended up with apoptosis. Treatment of these cells with melatonin could increase LC3-II levels, reduce Bax translocation to mitochondria and cytochrome c secretion from mitochondria to the cytoplasm, suggesting that melatonin enhanced autophagy and reduced apoptosis of SH-SY5Y cells. Further experiments showed that in the presence of autophagy inhibitors (such as bafilomycin A1, 3-MA, and ATG5 siRNA), the protective effect of melatonin would be weakened. This study showed that melatonin-induced autophagy protected SH-SY5Y cells from PrP-induced neurotoxicity [229]. Similarly, a recent study showed that melatonin prevented the PrP-mediated neurotoxicity to mitochondria by activating α-7 nicotinic acetylcholine receptors, and subsequently increased the occurrence of autophagy in PrP-exposed cells [230]. These studies showed that melatonin prevented neurotoxicity and autophagic cell death by acting as an autophagy activator.

At present, there are no relevant research reports on the effects of melatonin on other NDDs such as ALS and HD by regulating cell autophagy and its mechanism of protective action, although it has been reported that melatonin offers neuroprotection for ALS and HD by us and others [231,232,233,234]. The effects of melatonin on NDDs were not only mediated through regulating cell autophagy but also by the other mechanisms, as follows.

#### 4.2.2. Inhibition of Apoptosis

Cycle-dependent kinase 5 (CDK5) is important for the maintenance of neuronal function and plasticity. CDK5 imbalance exists in both PD and AD patients, especially accompanied by the increase of P25 [235]. Melatonin not only improves the viability of cells damaged by MPP^+^, but also inhibits its apoptotic effect. This anti-apoptotic effect is achieved by reducing the formation of CDK5/P25 fragments. Previous reports revealed that CDK5 could regulate the release of striatum dopamine and thus melatonin might also play a role in regulating the neurotransmitter dopamine [236,237]. Melatonin could inhibit UV-induced apoptosis of keratinocytes and human granulocyte monocyte leukemia U937 cells [238]. Melatonin with antioxidant property could also inhibit another important cell death signaling pathway enzyme c-Jun N-terminal kinase 1, which is usually stimulated by increased oxidative stress after exposure to potentially toxic substances [219,239]. Based on these results, the anti-apoptotic effects of melatonin are multi-faceted [231,233,234].

#### 4.2.3. Scavenging Free Radicals

Apoptosis and necrosis caused by excitatory toxins are prevalent in many NDDs. The main causes are calcium homeostasis and intracellular oxidative stress accompanied by ER stress and mitochondrial dysfunction. Melatonin can remove oxygen-free radicals and nitric oxide in the mitochondria, reduce the decrement of glutathione and oxidative protein damage in the mitochondria, and attenuate mitochondrial DNA damage. Melatonin also enhances the activity of mitochondrial electron transport chain complexes I and IV, thereby enhancing the rates of mitochondrial respiration and ATP synthesis during normal and oxidative stress [240,241]. MPTP-induced damage to the striatum and the midbrain dopamine system showed a decrease in dopamine level and an increase in hydroxyl radical (·OH), whereas MPTP-mediated changes could be reversed by melatonin supplementation in a dose-dependent manner [210,211,242]. Thomas et al. showed that melatonin could inhibit oxidative stress in MPTP-related oxidative damage models, both in vivo and in vitro [243]. Melatonin not only prevented the increase of ·OH in the substantia nigra and caudate nucleus of mice treated with MPTP/MPP^+^, but also increased the levels of reduced glutathione and SOD activity. No significant effect on MAO-B activity was observed [243]. Melatonin could reverse the MPTP-mediated suppressed activity of mitochondrial complex I and inhibit the autooxidation of dopamine, thereby reducing oxidative stress. At the same time, melatonin and its primary metabolite, 6-hydroxymelatonin, inhibited oxidative damage in the hippocampus caused by metal ions [244].

#### 4.2.4. Neuroprotection

Melatonin can exert neuroprotective effects on a variety of neuronal injuries by excitatory amino acids, Aβ, MPTP/MPP^+^, etc. For instance, the protective role of melatonin in glutamate-induced excitotoxicity can be good for not only NDDs but also for other neuronal injuries such as ischemia and epilepsy [234,245,246]. The protective mechanism might be related to the prolonged activation of the Akt signaling pathway. The Akt is a downstream effector molecule of phosphoinositide kinase-3 (PI3K) and plays an important role in promoting the survival of many damaged neurons. Phosphorylated Akt (p-Akt) further phosphorylates a variety of anti-apoptotic proteins to inhibit apoptosis [247]. Previous studies observed that the Akt was significantly phosphorylated 2 h after melatonin treatment, and its phosphorylation lasted for up to 24 h. The expression of glial-cell-line-derived neurotrophic factor (GDNF) in the CA3 area was significantly increased after 6 h of melatonin treatment. After 24 h, the GDNF was mainly secreted by astrocytes [248,249].

Therefore, activation of Akt could be achieved through two pathways: 1) Melatonin bound to MT1 and MT2 receptors on neurons, activated the insulin tyrosine receptor kinase, thereby activating PI3K/Akt; and 2) melatonin acting indirectly on astrocytes. Melatonin activated neurons to promote the secretion of GDNF by astrocytes, whereas GDNF bound to GDNF family receptor-1 (GF-1) stimulated tyrosine phosphorylation, and in turn activated the PI3K/Akt-signaling pathway.

#### 4.2.5. Neurotrophic Effect

A study showed that 10^−12^~10^−9^ mol/L melatonin increased the expression of GDNF mRNA and protein in a dose-dependent manner after rat C6 glioma cells were treated for 24 h, and this activation seemed to be mediated by a G protein-coupled receptor [250]. Lee et al. showed that in a mouse model damaged by kainic acid, melatonin pretreatment enhanced the release of GDNF from astrocytes. This promotion effect appeared at 6 h after administration and lasted until 24 h after post-treatment [248]. Some studies reported that in the PD model with unilateral damage of 6-OHDA, the expression of GDNF on the contralateral side increased in a compensatory manner. After melatonin treatment, the expression of contralateral GDNF decreased to a normal level and this may have represented one of the neuroprotective effects, although the specific mechanism of action is unknown. At the same time, the expression of the brain-derived neurotrophic factor (BDNF) was not significantly changed [251,252].

#### 4.2.6. Anti-Aβ Neurotoxicity

**Figure 1:**
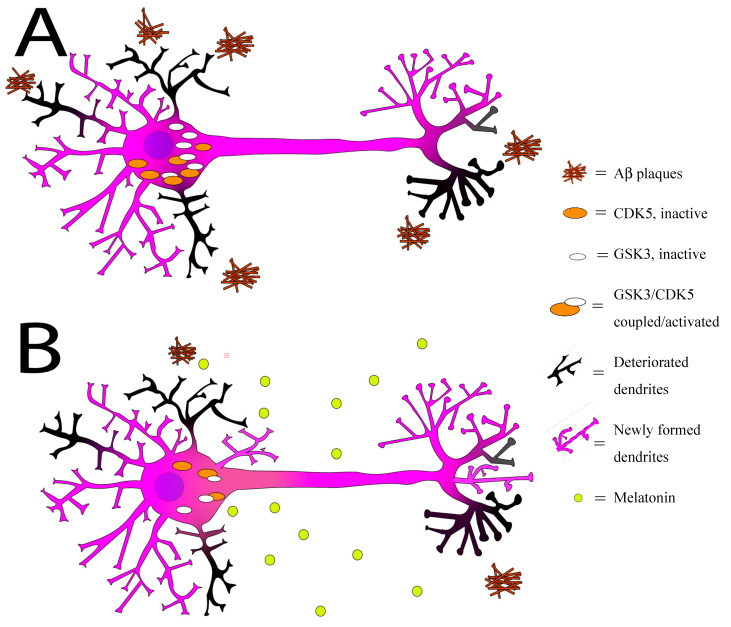
Melatonin regulates cycle-dependent kinase (CDK5) and glycogen synthase kinase 3 (GSK3), promotes dendritic growth of neurons, and flushes out Aβ plaques. (**A**) Neuron exhibits an excess of CDK-5 (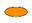), GSK3 (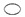), and A**β** plaques (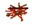) in the absence of melatonin. Some of the dendrites and axon terminals are already degrading (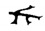). (**B**) Melatonin administration (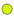) decreases CDK-5 and GSK3 accumulations, promotes growth of new dendrites and axon terminals (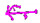), and flushes out some A**β** plaques.

Neurofibrillary tangles and vascular amyloidosis are characteristic pathological changes of AD in both human patients and experimental models. Aβ is the main component of SP and vascular amyloidosis. Melatonin could antagonize the accumulation of Aβ. Administration of melatonin could reduce the plaque formation in Aβ-expressed transgenic mice [253]. Melatonin also inhibited the release of extracellular vesicles (EVs), including exosomes in AD, thereby reducing the Aβ concentration and its toxicity. At the same time, it also affected the level of tau contained in EVs [251]. Experiments with the brains of the transgenic Drosophila overexpressed Aβ42 revealed that melatonin significantly improved the toxicity caused by Aβ42 overexpression [254]. Melatonin reduced tau hyperphosphorylation and Aβ accumulation by regulating the CDK5 signaling pathway and glycogen synthase kinase 3 (GSK3). Melatonin reduced the changes in circadian rhythm, blocked Aβ production, Aβ oligomerization and fibril formation, inhibited tau hyperphosphorylation, and decreased synaptic dysfunction, thus playing a protective role in the progression of AD [255] (Figure 1).

#### 4.2.7. Regulation of Neuroinflammation

Increased neuroinflammation is also involved in the initiation and progression of NDDs such as AD and PD. Melatonin could inhibit the activation of microglia and astrocytes [256,257], suppress activation of nuclear factor-κB (NF-κB), a critical transcription factor for many pro-inflammatory cytokines and chemokines, and thus attenuate the inflammatory process [258]. The basal level of the inflammatory response in the aging brain is continuously activated, but its ability to respond to acute inflammatory stress is decreased. Adding melatonin to the diet may reduce the level of the inflammatory response in the basal state and increase the response to acute inflammation induced by lipopolysaccharide. Other studies suggested that melatonin also regulates the body’s systematic inflammatory responses [185,259,260].

#### 4.2.8. Regulation of Cytoskeleton Proteins

Cytoskeleton proteins are important for maintaining the asymmetric morphology, polar structure, and normal physiological functions of neurons. NDDs are often accompanied by abnormal changes in cytoskeletal proteins, including tau protein, leading to some pathological deposits and damage to neurotransmitters. Melatonin could promote the growth of neurites by regulating the rearrangement of cytoskeletal proteins, suggesting that melatonin might clear pathological deposits in NDDs. Melatonin promoted dendritic maturation of neonatal neurons and the formation of synapses between axons and pyramidal neurons of neonatal neurons by regulating or restoring the cytoskeleton. Melatonin stimulated the structural plasticity of mossy fiber projections in the brain, thereby establishing functional synapses in the hippocampus. Melatonin also regulated the structural plasticity of axons in dentate gyrus granule cells in the brain [261]. Melatonin exhibited various beneficial effects on the development and physiology of CNS neurons. Melatonin could exert its function through two G protein-coupled membrane receptors (MT1 and MT2), regulating neurogenesis, synaptic function, neuronal cytoskeleton, and gene expression. It was also related to the expression of key markers of synaptic activity (synaptophysin, glutamate receptor 1, hemophilin, and glutamate decarboxylase 1) [262].

## 5. Conclusions

**Figure 2:**
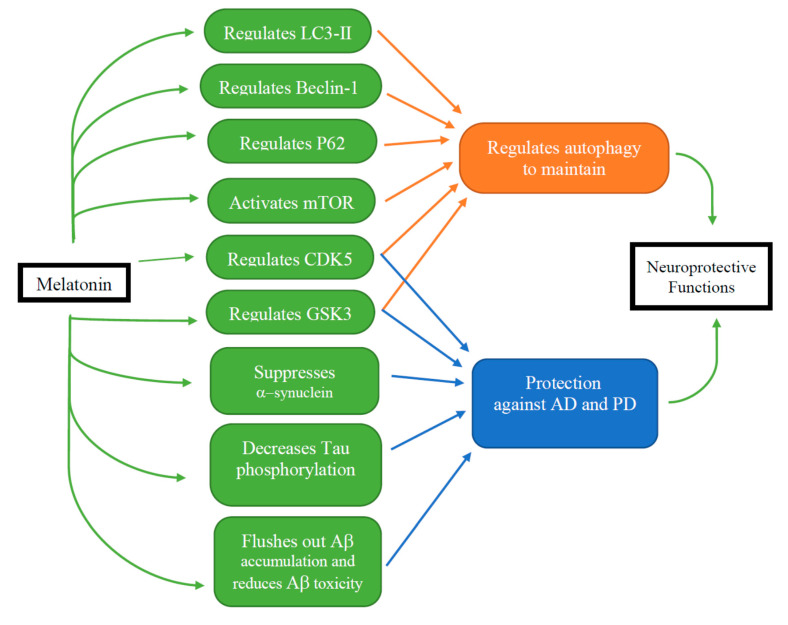
The multifaceted effects of melatonin. Melatonin regulates autophagy (via LC3-II, Beclin1, P62, and mTOR) as well as CDK5 and GDK3 to maintain cellular homeostasis. Neuroprotective mechanisms of melatonin include the decrease of tau phosphorylation, suppression of α-synuclein aggregation, flushing out of A**β** accumulation, and reduction A**β** toxicity in the experimental models of AD and PD.

This review briefly discussed the role of autophagy in neuronal cell death, focusing on the dual role of autophagy in neurons. There was no doubt that autophagy played an important role in the internal and external environments, balance and stability of cells and tissues, and the initiation and progression of degenerative diseases of the nervous system. As an important neuroprotective agent, melatonin can exhibit beneficial effects on various NDDs, suggesting that melatonin regulates the autophagy process through different mechanisms (Figure 2). Melatonin can exert its beneficial effects through either autophagy promotion and/or autophagy inhibition, depending on the cellular contexts, and change the nervous system through autophagy. Melatonin can also show potent antioxidant even in the mitochondria and anti-apoptotic effects. These properties support that melatonin is a safe and important protective agent in the CNS and shows beneficial effects on various NDDs. Melatonin plays a neuroprotective role in NDDs via regulating autophagy, which may be a newly emerging field. Finally, melatonin is a valuable agent for further translational research and clinical studies to discover agents to prevent and/or cure NDDs such as AD, PD, HD, OPIDN, and ALS.

## Figures and Tables

**Table 1:** The main functions of autophagy-related proteins.

Autophagy-Related Protein	Functions in Autophagy	References
ULK1 complex	Reduces phosphorylation, activates ULK1, ULK2, ATG13, ATG101, and FIP200 complexes Involved in induction	[34,35,36]
PI3K-III	VPS34, VPS5, AMBRA1, UVRAG, BIF1, ATG14, and Beclin1 complex Bcl-2 displacement activates VPS34 complex Participates in nucleation	[34,37,38,39]
ATG9	Transmembrane proteins, only found in phagocytosis Participates in nucleation	[34,40,41,42,43]
Beclin1/ATG6	Interacts with Bcl-2 Interacts with negative autophagy regulator Golgi-associated plant pathogenesis-related protein 1 (GAPR-1)	[39,44,45,46,47]
ATG7, ATG10	E1 and E2-like enzyme Participates in the conjugation of ATG12-ATG5	[34,48,49,50]
ATG4	Required for autophagic elongation	[34,51,52]
ATG3	Cysteine protease, cleaves LC3	[34,53,54]
P62/SQSTM1	Cellular content receptors that are preferentially degraded by autophagy	[55,56,57,58]
LC3/ATG8	The only autophagy-associated protein associated with autophagosome membranes	[35,43,59,60,61]

**Table 2:** Autophagy and various neurodegenerative diseases (NDDs).

NDDs	Autophagy Sites Associated with NDDs	Mechanism of Autophagy in NDDs	Changes in Autophagy	References
AD	PS1 and PS2 mutations	Impaired glycosylation Lysosomes damaged by acidification	Reduced autophagosomes degradation Accumulation of Aβ	[92,93,94,95]
	PICALM decreases or disappears	Impaired phagocytic elongation, autophagosomes formation, and autophagosome-lysosomal-fusion protein	Reduced autophagosomes degradation	[96,97,98,99]
	Mutation of amyloid precursor protein (APP)	Increased mTOR pathway	Autophagy inhibition Aβ and tau accumulation	[86,87]
	Aberrant expression of P62/SQSTM1	Hyperphosphorylated tau is associated with neurofibrillary tangles	Reduced autophagy of specific cytoplasm	[60,115,116,117,118,119]
	Beclin1 decreases	Defects in autophagosome synthesis	Reduced autophagosomes degradation Accumulation of Aβ	[100,101,102,103]
	mTOR activity is increased	Activity of autophagy reduces	Autophagy inhibition Aβ accumulation and hyperphosphorylated tau	[104,105,106,107,108,109,110,111,112,113]
	NRBF2 protein increases	Reduced expression of autophagy substrate SQSTM1	Reduced APP amyloidosis	[114]
PD	α-synuclein overexpression	ATG9 misalignment	Reduced macroautophagy	[133]
	α-synuclein mutations	Inhibition of CMA binding to chaperone molecule PARP activation	Autophagy inhibition, Lewy body formation Autophagy inhibition, α- synuclein increases	[124,126,129,130,134,135,136,137]
	Absence of Park2	LC3 II decreases	Downregulation of autophagy	[140]
	Beclin1	Beclin1 decreases	Increased protein aggregate	[148,150]
	PINK1/Parkin mutation	Mitochondrial protein ubiquitin labeling reduced	Reduced autophagy in damaged mitochondria	[137,138]
	mTOR increases	AMPK/ mTOR-mediated autophagy/apoptosis pathway	Reduced autophagy, inhibition of MPTP induced neuron death	[146,147,148,149]
HD	mHTT	Reduced antagonism to mTOR pathway	Inhibited autophagy	[157,158]
	Beclin1	mHTT and Beclin1 bind nonspecifically and consume Beclin1, Beclin1 level decreased	Reduced autophagosome formation and impaired autophagy	[160]
	Reduced ALFY mRNA expression	Reduced ALFY expression	Reduced degradation of mHTT, impaired autophagy	[162,163,164]
	Phosphorylation of p62/SQSTM1	p62/SQSTM1 and ULK1 interact with each other	Increased protein aggregate clearance, impaired autophagy	[156,161]
OPIDN	Beclin1	-	Impaired autophagy, aggregation of mitochondria and vesicles	[169]
ALS	SOD1 mutation	Function loss of dynein	Reduced autophagy, increased protein aggregation	[175,183]
	Gene mutation	Interact with LC3	Increased autophagosome and autophagic matrix binding	[178,179,180]
	OPTN	Reduced clearance of damaged mitochondria	Reduced autophagy, increased protein aggregation	[181,182]

## Abbreviations

Aβ	Amyloid-β
AD	Alzheimer’s disease
Akt	serine/threonine kinase 1
ALFY	autophagy-related FYVE protein
ALS	amyotrophic lateral sclerosis
AMBRA1	autophagy and beclin1 regulator 1
AMPK	AMP-activated protein kinase
APP	amyloid precursor protein
ATG	autophagy-related gene
ATG14L	ATG14-like
BAX	Bcl-2-associated X protein
BDNF	brain-derived neurotrophic factor
Beclin1	Bcl2 interacting protein
BECN1	beclin1
BIF1	Bax-interacting factor-1
CDK5	cyclin-dependent kinase 5
CMA	chaperone-mediated autophagy
CNS	central nervous system
COX-2	cyclooxygenase-2
E1-	like enzyme
UBA5	ubiquitin like modifier activating enzyme
ERK1 / 2	extracellular signal-regulated kinases 1/2
FIP200	focal adhesion kinase family interacting protein of 200kD
GAPR-1	Golgi-associated plant pathogenesis-related protein 1
GDNF	glial-cell-line-derived neurotrophic factor
GSK3	glycogen synthase kinase 3
HD	Huntington’s disease
HTT	Huntington
JNK1/2	c-Jun N-terminal kinase 1/2
·OH	hydroxyl radical
6-OHDA	6-hydroxydopamine
LC3	microtubule-associated protein 1 light chain 3
MAO-B	monoamine oxidase B
METH	methamphetamine
mHTT	mutant Huntington
MPPP	1-methyl-4-phenyl-4-propionoxypiperidine
MPTP	methyl-4-phenyl-1,2,3,6-tetrahydropyridine
mTOR	mammalian rapamycin target protein
NDDs	neurodegenerative diseases
NF-κB	nuclear factor-κB
NRBF2	nuclear receptor binding factor 2
OPIDN	organophosphate-induced delayed neuropathy
OPTN	optineurin
Parkin	RING-domain-containing E3 ubiquitin ligase
PARP	poly-ADP ribose polymerase
PD	Parkinson’s disease
PI3KCIII	phosphatidylinositol 3-kinase catalytic subunit type 3
PI(3)P	phosphatidylinositol 3-phosphate
PICALM	phosphatidylinositol-binding clathrin assembly protein
PINK1	PTEN-induced kinase 1
PrP	viral proteins
PS	presenilin
PtdIns3K	class III phosphatidylinositol 3-kinase complex
RB1CC1	RB1-coiled helix-1
ROS	reactive oxygen species
SNARE	soluble NSF attachment protein receptor
SOD	superoxide dismutase
SP	senile plaque
SQSTM1	sequestosome 1
STX17	synaptic fusion protein-17
TFEB	transcription factor EB
TOCP	tri-ortho-cresyl phosphate
ULK1	Unc-51-like kinase 1
UVRAG	ultraviolet irradiation resistance-associated gene
Vps	vesicular protein-sorting protein
Vps34	type III PI3K
